# Messy eaters: Swabbing prey DNA from the exterior of inconspicuous predators when foraging cannot be observed

**DOI:** 10.1002/ece3.4866

**Published:** 2019-01-15

**Authors:** Ryan P. Bourbour, Breanna L. Martinico, Megan M. Crane, Angus C. Hull, Joshua M. Hull

**Affiliations:** ^1^ Department of Animal Science University of California, Davis Davis California; ^2^ Golden Gate Raptor Observatory San Francisco California

**Keywords:** DNA barcoding, foraging ecology, migration ecology, predator–prey interactions, raptor

## Abstract

Complex coevolutionary relationships among competitors, predators, and prey have shaped taxa diversity, life history strategies, and even the avian migratory patterns we see today. Consequently, accurate documentation of prey selection is often critical for understanding these ecological and evolutionary processes. Conventional diet study methods lack the ability to document the diet of inconspicuous or difficult‐to‐study predators, such as those with large home ranges and those that move vast distances over short amounts of time, leaving gaps in our knowledge of trophic interactions in many systems. Migratory raptors represent one such group of predators where detailed diet studies have been logistically challenging. To address knowledge gaps in the foraging ecology of migrant raptors and provide a broadly applicable tool for the study of enigmatic predators, we developed a minimally invasive method to collect dietary information by swabbing beaks and talons of raptors to collect trace prey DNA. Using previously published COI primers, we were able to isolate and reference gene sequences in an open‐access barcode database to identify prey to species. This method creates a novel avenue to use trace molecular evidence to study prey selection of migrating raptors and will ultimately lead to a better understanding of raptor migration ecology. In addition, this technique has broad applicability and can be used with any wildlife species where even trace amounts of prey debris remain on the exterior of the predator after feeding.

## 1 INTRODUCTION

Foraging ecology and predator–prey interactions have shaped the natural histories of species, including distribution and abundance as well as complex behaviors such as foraging strategies, interspecies competition, and timing and route of migration (Abrams, 2000; Alerstam, Hedenström, & Åkesson, 2003). Even during migration, birds must feed along their migratory route, creating ephemeral dynamics between migratory predators and prey (Ydenberg, Butler, & Lank, 2007). Diurnal birds of prey, for example, accipiters and falcons, use powered flight during migration and must continuously hunt en route to meet high energetic demands (DeLong & Hoffman, 2004; Kerlinger, 1989). To meet these energetic requirements, raptors are thought to time migration to track migratory avian prey species while some avian prey are thought to time migration to avoid the influx of predators (Aborn, 1994; Ydenberg et al., 2007).

Determining the true role predator–prey interactions play on shaping migration strategies is difficult without accurate dietary information. To date, literature on the diet of migrating raptors that feed en route is primarily based on opportunistic observations and correlations between peak movement of predator and prey migrants (Aborn, 1994; Nicoletti, 1997; Ydenberg et al., 2007). Currently, ecologists lack tools that can be utilized to study the diet of raptors while migrating, as traditional methods often fall short (e.g., observations, nest cameras, prey remains, pellets, fecal, and stable isotopes; Marti, Bechard, & Jacksic, 2007).

**Figure 1:**
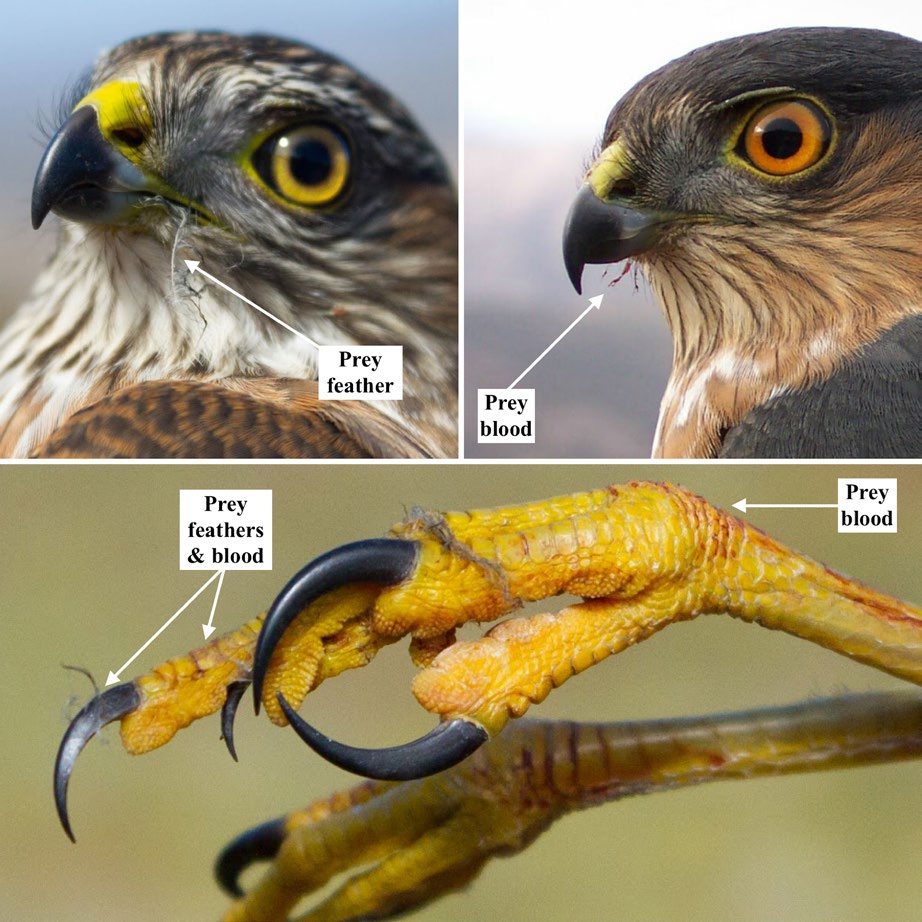
Close‐up images of migrating sharp‐shinned hawk beaks and talons. Visible prey feathers and blood are good indicators that prey DNA remains from a previous meal. Raptors, such as accipiters and falcons, will hunt daily during migration; therefore, prey DNA on beaks and talons can shed light on what fuels raptor migration and may reveal predator–prey interactions between bird‐eating raptors and songbird migrants within a migratory flyway. (Top right photo: Siobhan Ruck)

Limitations to diet studies of migrating raptors, and other enigmatic predators, may be alleviated by sampling molecular residues of prey remains from the exterior of beaks and talons (Figure 1). Prey DNA can then be referenced to cytochrome oxidase 1 (COI) gene sequences, or other appropriate markers, that are unique to the species level (genetic barcodes) and have been previously cataloged in public barcode databases (Kerr et al., 2009). DNA metabarcoding has been a revolutionary tool in studying the diet of many wildlife species utilizing fecal or gut samples (Clare, 2014; Kress, García‐Robledo, Uriarte, & Erickson, 2015; Pompanon et al., 2012), but has yet to be implemented for raptor diet studies or by sampling the exterior of a predator's mouth and claws.

The benefit of this tool resides in the ability to document prey selection of wildlife when traditional methods are not possible and has the potential to be utilized for any wildlife species where even trace amounts of prey debris remain on the exterior of the predator after feeding, for example, vultures, piscivorous birds, insectivorous or predatory songbirds, and even nectarivores such as bats and hummingbirds, where trace plant DNA from pollen may be present (Nagarajan, Prabhu, Kamalakkannan, & Sinu, 2018). Gathering DNA from the exterior of the body can be a viable alternative to fecal sampling, where prey DNA may be highly degraded or in low quantities compared to predator DNA (King, Read, Traugott, & Symondson, 2008; O'Rorke, Lavery, & Jeffs, 2012), or when fecal sampling is not possible. For example, exterior swabbing can minimize handling time and stress compared to fecal sampling, which is a critical consideration for raptor research (Heath, 1997).

In North America, thousands of raptors are banded at monitoring stations situated along migration corridors, which offers a valuable opportunity to study the diet of migrating raptors and test novel methods for identifying prey species through collection of trace DNA. Our objectives are to (a) develop a minimally invasive method for use in studying the diet of predators using raptors as a case study; (b) verify that prey DNA can be successfully obtained and identified from raptors with a known diet; and (c) apply our method to wild migrating raptors and identify prey to species.

## 2 MATERIALS AND METHODS

### 2.1 Sampling method

**Figure 2:**
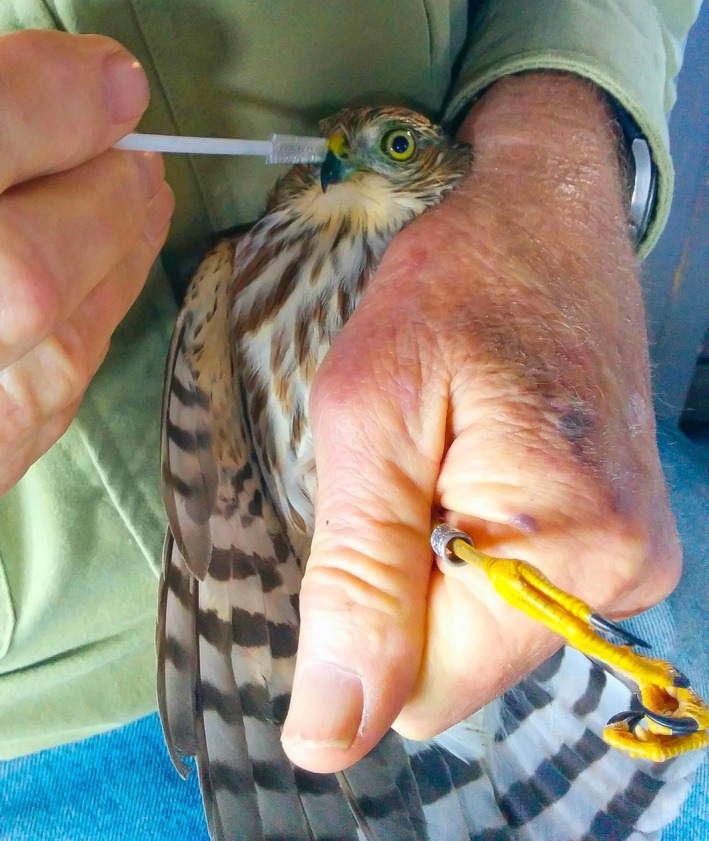
A juvenile migrating sharp‐shinned hawk having its beak swabbed for prey DNA after the banding process at a migration monitoring station. (Photo: Laura Young)

**Figure 3:**
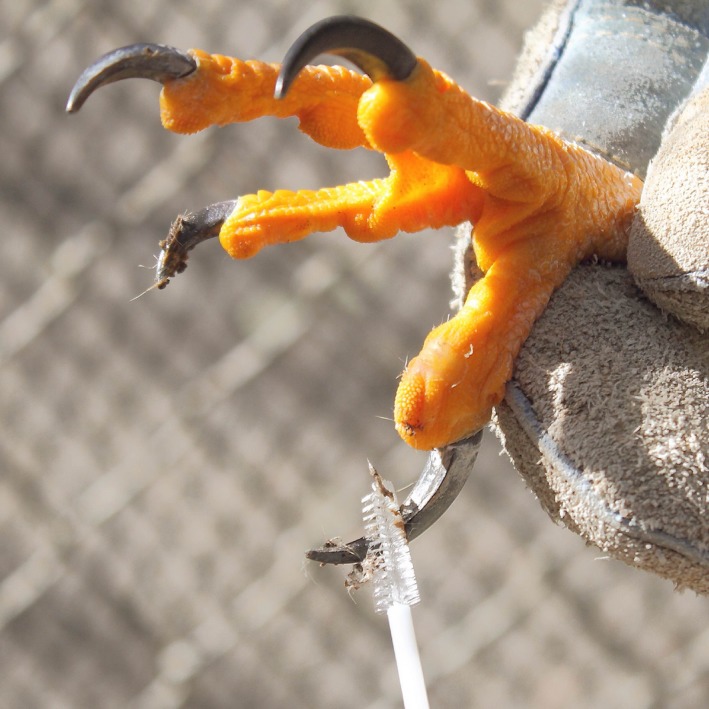
A captive raptor having its talons swabbed for prey DNA as part of our controlled study

For each raptor, we swabbed beaks and talons separately to (a) determine differences in DNA detectability and (b) as a precaution for PCR inhibitors that may be present on talons which come into contact with a variety of substrates. We first moistened nylon swab bristles (#25‐2188 Puritan Medical Products Company) in 0.7 ml ultrapure water. To sample beaks, we gently and thoroughly swabbed the entire exterior of the upper and lower mandible, targeting any visible prey blood or tissue if present (Figure 2). Precaution was taken to avoid contact between the swab and any interior mouthparts to prevent collecting predator DNA. To sample talons, we swabbed the entire surface of each talon, targeting visible prey blood, tissue, or feathers if present (Figure 3). Toe pads or scales were only swabbed if visible prey remains were present. For each sample collected, the nylon brush tip was removed and placed into individual 1.5 ml screw‐top centrifuge tubes containing 0.7 ml Longmire's lysis buffer (100 mM Tris pH8.0, 100 mM EDTA, 10 mM NaCl, 0.5% SDS, 0.2% sodium azide) and stored at −20°C.

### 2.2 DNA extraction and quantification

We extracted DNA from each brush tip using the QIAamp DNA Mini Kit (QIAGEN Inc.) with a modified protocol. After 20 µl proteinase K and 600 µl buffer AL was added, vortexed, and incubated for 15‐min, all liquid was transferred from the 1.5 ml screw‐top centrifuge tube to a 2.0 ml safe‐lock centrifuge tube to allow space for 600 µl of 100% ethanol. Following buffer washes, DNA was eluted into 30 µl of molecular grade H_2_O twice (60 µl H_2_O total). The purpose of using H_2_O for DNA elution is to have the option to increase DNA concentrations via evaporation. We quantified DNA concentration of each sample using Qubit dsDNA BR Assay Kit and 2.0 µl of DNA. We conducted all laboratory work in the Genomics Variation Laboratory (University of California, Davis).

### 2.3 Controlled study

To validate swabbing methods, we sampled three resident raptors at the California Raptor Center in Davis, California, USA. Each raptor had a diet of (a) mice (*Mus musculus*) only, (b) hatchling chickens (*Gallus gallus*) only, or (c) both. Feedings occurred between 08:00 and 09:00 every morning and sampling occurred between 14:00 and 16:00. Exact time of meal consumption was not documented; however, meals were completely or partially eaten prior to sampling. We sampled each raptor three times every other week. We tested for the presence of chicken DNA using a previously published chicken primer that targeted a 133‐bp amplicon (Dooley, Paine, Garrett, & Brown, 2004; Chicken forward: 5′–AGCAATTCCCTACATTGGACACA–3′; Chicken reverse: 5′‐GATGATAGTAATACCTGCGATTGCA–3′). We did not test for mouse DNA because we could not control for mice entering enclosures where captive raptors have been documented eating pest rodents.

### 2.4 Field study

We swabbed migrating sharp‐shinned hawks (*Accipiter striatus*; *n* = 285) and merlin (*Falco columbarius*; *n* = 41) during fall of 2015 that were trapped by the Golden Gate Raptor Observatory in the Marin Headlands, CA, USA. All individual raptors were trapped by dho‐ghazzas, which are passive nets that collapse upon impact, making contact with trap bait unlikely. We checked for the presence of songbird prey DNA in a random subset of the wild samples with DNA quantities >2.0 µg/ml using a previously published bird primer (González‐Varo, Arroyo, & Jordano, 2014; COI‐fsdF: 5′–GCATGAGCCGGAATAGTRGG‐3′; COI‐fsdR: 5′–TGTGAKAGGGCAGGTGGTTT‐3′) and used DNA extracted from songbird tissue samples obtained from the UC Davis Museum of Wildlife & Fish Biology as controls. Primers were ordered with barcode sequences attached to both forward (ACTG) and reverse (ATGCTAA) COI‐fsd primers consistent with the first round of PCR during library preparation for high‐throughput sequencing for DNA metabarcoding (Vo & Jedlicka, 2014). We prepared and sent DNA sequences to the Genome Center at the University of California, Davis for Sanger sequencing. We used a standard nucleotide BLAST search to reference all barcode sequences.

## 3 RESULTS

### 3.1 Controlled study

Quantifiable (>1.0 µg/ml) DNA was detected on all swabs collected from captive raptors. DNA concentrations for the “mouse only” raptor from talon swabs ranged from 5.0–16.0 µg/ml and beak swabs ranged from 1.31–4.24 µg/ml, with no chicken DNA detected on any swab. DNA concentrations for the “chicken only” raptor from talon swabs ranged from 5.20–55.6 µg/ml and beak swabs ranged from 1.65–2.37 µg/ml with all swabs testing positive for chicken DNA. DNA concentrations for the “both mice and chicken” raptor from talon swabs ranged from 39.3–171.0 µg/ml and from beak swabs ranged from 2.15–3.24 µg/ml with chicken DNA detected on all talon swabs and only 1 (3.24 µg/ml DNA) of 3 beak swabs.

### 3.2 Field study

Out of 285 sharp‐shinned hawks and 41 merlins sampled, we obtained quantifiable (>1.0 µg/ml) DNA concentrations (potential dietary data) from 205 (71.4%) and 40 (97.6%) individuals, respectively. Out of the 205 sharp‐shinned hawk individuals, 191 talon (92.7%) and 100 beak (48.5%) swabs had quantifiable DNA concentrations, and we detected songbird DNA on all samples: 9 talon and 1 beak (Table 1). Out of the 41 merlin individuals, 37 talon (90.2%) and 35 beak (85.4%) swabs had quantifiable DNA concentrations, and we detected songbird DNA on all samples: 4 talon and 5 beak (Table 1). The top match for all COI sequences was probable prey in the sampling area (Table 1; Supporting Information Table S1).

**Table 1:** Results from Sanger sequencing a random subset of samples collected from beaks and talons of migrating sharp‐shinned hawks and merlins

Species	Sample	µg/ml	Trap/Lure	Crop	Prey	*E* value	% Match
Sharp‐shinned Hawk	Talon	28.8	DG/ST	f	Fox Sparrow (*Passerella iliaca*)	0	95
		18.7	DG/ST	f	Townsend's Warbler (*Setophaga townsendi*)	5 × 10^−179^	95
		13.8	DG/ST	f	Fox Sparrow (*Passerella iliaca*)	2 × 10^−95^	94
		14.6	DG/ST	f	American Goldfinch (*Spinus tristis)*	0	99
		33.8	DG/ST	f	California Thrasher (*Toxostoma redivivum*)	0	95
		4.61	DG/ST	f	Swainson's Thrush (*Catharus ustulatus*)	0	96
		6.11	DG/ST	e	Red‐breasted Nuthatch (*Sitta canadensis*)	0	98
		7.21	DG/HS	f	Red‐breasted Nuthatch (*Sitta canadensis*)	3 × 10^−127^	88
		24.5	DG/ST	f	Dark‐eyed Junco (*Junco hyemalis*)	0	99
	Beak	30.2	DG/ST	e	California Towhee (*Melozone crissalis*)	0	99
Merlin	Talon	2.24	DG/HS	e	Red‐breasted Nuthatch (*Sitta canadensis*)	7 × 10^−173^	99
		62.8	DG/HS	e	Red‐breasted Nuthatch (*Sitta canadensis*)	0	99
		8.07	DG/ST	e	Yellow Warbler (*Setophaga petechia*)	6 × 10^−153^	95
		3.44	DG/ST	e	European Starling (*Sturnus vulgaris*)	4 × 10^−175^	95
	Beak	13.26	DG/HS	e	Varied Thrush (*Ixoreus naevius*)	4 × 10^−160^	95
		2.04	DG/HS	e	House Sparrow (*Passer domesticus*)	0	99
		4.14	DG/HS	e	House Finch (*Haemorhous mexicanus*)	6 × 10^−158^	97
		3.03	DG/ST	e	Yellow Warbler (*Setophaga petechia*)	2 × 10^−173^	97
		2.83	DG/ST	e	American Robin (*Turdus migratorius*)	0	99
Control	SWTH	5.0	—	—	Swainson's Thrush (*Catharus ustulatus*)	0	98
	OCWA	5.0	—	—	Orange‐crowned Warbler (*Vermivora celata*)	0	98

Note

Presented are DNA concentrations (µg/ml) obtained from nylon brush tip, the trap (dho‐ghazza = DG) and bait (European starling = ST; house sparrow = HS) used to catch raptor, the status of crop upon capture (empty = e; full = f), and the species that COI sequence most closely aligned with using BLAST search tool (*E* Value = likelihood match is by chance; %Match = percentage of nucleotides aligned). Refer to Supporting Information Table S1 for COI sequences used to match species with BLAST search.

## 4 DISCUSSION

We successfully developed and tested a minimally invasive tool to document the diet of migrant raptors, and other enigmatic predators, by swabbing beaks and talons. We demonstrated that prey DNA can successfully be collected and identified from the exterior of a predator even when a recent feeding was not evident and visible prey remains were not present. Importantly, this swabbing method can be used to study more than diet during raptor migration, a life history stage where foraging ecology has never been systematically studied; it can also be applied to other wildlife species in studies with various objectives, such as those pertaining to food web dynamics, foraging ecology, predator–prey interactions (DeLong, Cox, Cox, Hurst, & Smith, 2013; Kress et al., 2015; Nagarajan et al., 2018; Pompanon et al., 2012), or even studies linking diet to microbiota (McFall‐Ngai et al., 2013).

Molecular markers should be selected appropriately for prey species groups, such that previous knowledge of the probable prey is necessary; novel or rare prey species can still be detected if the DNA can be targeted with primers in situ and amplified with PCR (Pompanon et al., 2012). In this study, we targeted the COI gene because sequences are well represented and cataloged for songbirds and can give resolution between closely related species (Kerr et al., 2009; Patel, Waugh, Millar, & Lambert, 2010). Prey DNA may be subject to differential degradation rates due to external environmental factors. To account for this, multiple primer sets may be used to reconstruct the marker region if DNA is found to be highly degraded (Patel et al., 2010). If prey DNA is not cataloged publicly, a reference library can be developed by sequencing potential prey DNA at the marker selected, but breadth of diet needs to be taken into account in order to create a thorough reference library (DeLong et al., 2013).

We only detected probable songbird prey on migrating raptors; however, two of the sequences matched bird species used to bait traps that are also common prey in the wild, which should be taken into account in dietary analyses when species are caught with baited traps. We determined that beaks and talons can be swabbed together to increase likelihood of collecting prey DNA, as both sample types contained prey DNA that was able to be amplified and sequenced. Although, it is not necessary to sample both beaks and talons if there are limitations due to predator's life history, for example, an owl roosting on feces and regurgitated pellets from other individuals might only have its beak swabbed. We did not detect predator DNA on swabs, so blocking primers were not necessary (O'Rorke et al., 2012), even when using primers to detect prey from the same class (Aves). For studies including a higher quantity of samples with potentially multiple prey species per sample, DNA metabarcoding using high‐throughput sequencing may be more appropriate and economical than Sanger sequencing (Vo & Jedlicka, 2014). Lastly, all biases associated with using DNA metabarcoding for dietary analyses should be considered (Deagle et al., 2018).

## CONFLICT OF INTEREST

None declared.

## AUTHORS’ CONTRIBUTIONS

JMH conceptualized method. RPB and JMH designed the study. ACH, RPB, and BLM coordinated permissions and sample collection. RPB and MMC designed and conducted laboratory work. RPB managed and analyzed data. RPB and BLM led manuscript writing. All authors contributed critically to manuscript and gave final approval for submission and declare no conflicts of interest.

## Data Availability

The authors agree to submit the data supporting the results of this study upon publication using Dryad.
