# Supplementary material for: Messy eaters: Swabbing prey DNA from the exterior of inconspicuous predators when foraging cannot be observed
Source: Ecol Evol. 2019 Jan 15;9(3):1452–7. doi: 10.1002/ece3.4866 (PMC6374680; doi:10.1002/ece3.4866)
Supplement: Supplementary file 1 [file ECE3-9-1452-s001.docx]

| **Sample ID** | **Raptor** | **Recovered COI Sequence** | **Prey** | **BLAST Accession #** |
| --- | --- | --- | --- | --- |
| 2015-217 | SSHA | CGGCAGACTGGGCCACCTGGCGCCCTTCTAGGAGACGACCAAGTCTATAACGTAGTCGTCACAGCTCATGCTTTCGTAATAATCTTCTTTATAGTTATGCCAATCATAATCGGAGGATTCGGAAACTGACTAGTTCCCCTAATAATTGGAGCCCCGGACATAGCATTCCCACGAATAAACAACATAAGCTTCTGACTTCTTCCCCCATCCTTCCTACTCCTCTTAGCATCCTCTACTGTTGAAGCAGGCGTCGGAACAGGCTGAACAGTGTACCCCCCACTAGCAGGCAACCTGGCCCACGCCGGAGCCTCAGTCGATCTTGCAATCTTCTCCCTACACCTAGCCGGTATCTCTTCAATCCTAGGAGCAATCAACTTCATTACAACAGCAAATTAATATGAAACCACCTGCCTTATCACTTTAACCATA | *Passerella iliaca* | HM033630.1 |
|  |  |  |  |  |
| 2015-184 | SSHA | AGGCCACCCGGCGCCCTTCTGGGAGACGACCAAGTCTACAACGTAGTCGTCACGGCCCATGCCTTCGTAATAATTTTCTTTATAGTTATGCCAATTATGATCGGAGGATTCGGAAACTGACTAGTCCCACTAATAATCGGAGCCCCAGACATAGCATTCCCACGAATAAACAACATAAGCTTCTGACTACTCCCACCATCATTCCTTCTTCTCCTAGCATCCTCCACAGTAGAAGCCGGAGCAGGAACAGGATGAACCGTGTACCCCCCACTAGCTGGCAACCTAGCCCATGCCGGAGCTTCAGTCGACCTTGCTATTTTCTCTTTACACTTAGCCGGAATTTCCTCAATCCTAGGGGCGATCAACTTCATTACTACAGCAATTAACATGAAACCACCTGCCCTATCA | *Setophaga townsendi* | HM033442.1 |
|  |  |  |  |  |
| 2015-192 | SSHA | CCGGCAGACTAGGCCACCATGCAGCCCTCTCTGGGAGACGACCAAGTCTATAACGTAATCGTCACGGCCCATGCTTTCGTAATAATCTTTTTTATAGTTATGCCAATTATAATCGGAGGATTCGGAAACTGACTAGTTCCCCTAATAATTGGAGCCCCGGACATAGCATTCCCACGAATAAACAATATAAGCTTCTGACTACTACCCCCATCCTTCCTACTCCTCCTAGCATCCTCTACTG | *Passerella iliaca* | JN850753.1 |
|  |  |  |  |  |
| 2015-010 | SSHA | CAGCCCCTAGCCTTCTCTCCGGCAGACTAGGTCAACCCGGAGCCCTCCTAGGAGACGACCAAGTCTACAACGTAATCGTCACGGCCCATGCTTTCGTTATAATCTTCTTCATAGTTATACCCATCATAATCGGAGGATTCGGAAACTGACTAGTTCCTCTAATGATCGGAGCCCCAGACATAGCATTCCCACGAATAAATAACATAAGCTTCTGACTACTTCCCCCATCATTCCTCCTCCTACTAGCATCTTCCACCGTAGAAGCAGGTGTTGGTACAGGCTGAACAGTATACCCTCCACTAGCTGGTAACCTAGCTCATGCCGGAGCTTCAGTTGACTTAGCAATTTTCTCCCTACACTTAGCCGGTATCTCTTCAATCCTAGGCGCAATCAACTTCATTACAACAGCAATCAATATAAAACCACCTGCCCTATCCTT | *Spinus tristis* | FJ236301.1 |
|  |  |  |  |  |
| 2015-107 | SSHA | GGGAAACCCTGGGAGCCCCTTCCTAGGCAGACGACCAAGTATTAAAATGTAGTCGTCACTGCCCATGCTTTCGTAATAATCTTCTTTATAGTTATGCCAATTATGATCGGAGGGTTTGGAAACTGACTAGTCCCCCTAATAATTGGAGCCCCAGACATAGCATTCCCACGAATAAATAATATGAGCTTCTGACTGCTACCCCCATCCTTCCTGCTACTCCTGGCATCCTCCACCGTAGAATCAGGAGCAGGAACAGGCTGAACCGTGTACCCACCTCTAGCCGGCAACCTAGCCCACGCTGGAGCTTCCGTAGACCTAGCCATCTTTTCCCTGCATCTAGCTGGCATTTCTTCCATCCTAGGAGCCATTAACTTTATTACAACAGCAATTAACATAAAACCACCTGCCCTATCCTT | *Toxostoma redivivum* | JN806017.1 |
|  |  |  |  |  |
| 2015-271 | SSHA | CCATGCACCTAGCCTCCTCTCCGGCCGAACTAGGCCACCCGGAGCCCTCCTGGGAGACGACCAAGTTTACAACGTAGTCGTCACGGCCCATGCTTTCGTGATAATCTTCTTCATAGTTATACCTATTATAATCGGAGGATTTGGAAACTGACTAGTCCCCCTAATAATCGGAGCCCCAGACATAGCATTCCCACGAATAAACAACATAAGCTTCTGACTACTTCCCCCATCCTTCCTACTCCTCCTAGCATCTTCTACCATTGAAGCAGGTGTCGGCACAGGCTGAACAGTATACCCCCCACTTGCCGGCAACCTAGCACACGCTGGAGCCTCAGTCGATCTCGCAATTTTCTCTCTACACCTAGCCGGTATCTCTTCAATCCTAGGAGCAATCAACTTCATCACAACAGCAGTCAACATGAAACCACCTGCCCTATCACTTAAGCATA | *Melozone crissalis* | DQ433097.1 |
|  |  |  |  |  |
| 2015-101 | SSHA | CCTGCTCTAGCCTTCTCTTCCGGCAGACTAGGCCACCCGGCGCACCTACCTAGGGAGACGACCAAATCTACAATGTAGTTGTCACCGCCCATGCCTTCGTAATGATTTTCTTTATAGTTATGCCAATCATGATTGGGGGGTTCGGAAACTGGCTAGTCCCATTAATAATCGGAGCCCCAGACATAGCATTCCCCCGAATAAACAACATAAGCTTCTGACTTCTCCCACCATCATTCCTCCTTCTCCTAGCCTCCTCCACAGTAGAAGCAGGAGCAGGAACAGGATGAACCGTGTACCCACCCCTAGCTGGCAACCTAGCACACGCAGGAGCTTCAGTCGACCTAGCTATTTTCTCCCTACACTTAGCAGGAATCTCCTCAATCCTAGGGGCCATCAACTTCATTACTACAGCAATCAACATAAAACCACCTGCCCTATCAATAA | *Catharus ustulatus* | HM033291.1 |
|  |  |  |  |  |
| 2015-171 | SSHA | CAGCACCTAGTCCACTTTTCCGGCAGACTAGGCCACCAGGCGCCCTCTTGGGAGACGACCAAGTATATAACGTAATCGTCACGGCCCATGCTTTCGTAATAATCTTTTTTATAGTTATGCCAATTATGATTGGAGGATTTGGAAACTGACTAGTTCCTCTAATAATTGGAGCACCTGACATAGCATTCCCACGAATGAACAACATAAGCTTCTGACTTCTACCCCCATCCTTTCTTCTCCTACTAGCCTCCTCTACAGTAGAGGCCGGAGTAGGAACAGGATGAACTGTGTATCCTCCCCTGGCTGGTAATTTAGCTCACGCCGGGGCGTCAGTTGATTTAGCAATTTTCTCCCTACATCTAGCAGGAATTTCATCTATCCTAGGGGCAATCAATTTCATTACCACTGCAATTAACATAAAACCACCTGCCCTATCAATTAGCATA | *Sitta canadensis* | DQ434090.1 |
|  |  |  |  |  |
| 2015-073 | SSHA | CTAGGCCACCAGGCGCCCTCTTGGGAGACGACCAAATTACAACGTAATCGTCACGGCCCATGCTTTCGTAATAATCTTTTTTATAGTTATGCCAATTATGATCGGAGGATTCGGAAACTGACTAGTTCCTCTAATAATTGGAGCCCCTGACATAGCATTCCCACGAATGAACAACATAAGCTTCTGACTTCTACCCCCATCCTTTCTTCTTCTACTAGCCTCCTCCACAGTAGAGGCAGGAGCAGGAACAGGATGAACTGTGACCCTCCCCCTGCTGGCAACCTTACCCACGCCCGAGCCGCCGTATACCTGGCTATCTTTTTCCTTACATTAACCAGGATTTTCTCATATCTAAGGGCCATCAACTTCATTTCCCCCTGAATTAAAATAAAAACACCTGGCCTTTTACC | *Sitta canadensis* | JN850725.1 |
|  |  |  |  |  |
| 2015-100 | SSHA | AGCCCTCCTAGGAGACGACCAAGTCTATAACGTAGTCGTCACAGCCCACGCCTTCGTAATAATCTTCTTCATAGTTATACCAATTATAATCGGAGGATTTGGAAACTGACTAGTTCCACTAATAATCGGAGCCCCGGACATAGCATTCCCGCGAATAAATAACATAAGTTTTTGACTACTCCCCCCATCCTTTCTCCTCCTCCTAGCATCCTCTACCATTGAAGCAGGTGTCGGCACAGGCTGAACAGTATACCCCCCACTAGCAGGCAACCTAGCCCACGCTGGAGCCTCAGTCGACCTCGCAATCTTCTCCCTACACTTAGCCGGCATCTCCTCAATCCTAGGGGCCATCAACTTCATCACAACAGCAATCAACATAAAACCACCTG | *Junco hyemalis* | KX461113.1 |
|  |  |  |  |  |
| 2015-228 | MERL | GTAGTCGTCACCGCCCACGCTTTTGTCATAATCTTCTTCATAGTAATGCCAATCATAATCGGGGGGTTCGGAAACTGACTGGTGCCCTTAATAATTGGAGCTCCAGACATAGCCTTCCCCCGAATAAACAACATGAGCTTCTGACTACTTCCCCCATCCTTCCTACTACTATTAGCCTCCTCCACAGTAGAAGCAGGGGCGGGAACAGGATGAACCGTCTATCCCCCCCTAGCCGGAAACCTAGCACATGCAGGAGCCTCAGTGGACCTAGCTATTTTTTCCCTACACCTAGCAGGGATTTCCTCAATCCTGGGGGCTATCAACTTCATTACTACAGCAATTAACATAAAACCACCTGCCCTATC | *Ixoreus naevius* | JN850755.1 |
|  |  |  |  |  |
| 2015-237B | MERL | GGAGATGACCAAGTTTACAACGTAGTTGTCACAGCCCATGCTTTCGTGATAATCTTCTTTATAGTTATGCCAATTATAATTGGGGGATTCGGAAACTGACTAGTCCCACTGATAATTGGAGCACCAGACATAGCATTCCCACGAATAAACAACATAAGCTTCTGACTGCTACCCCCATCCTTCCTCCTGCTACTAGCATCCTCCACCGTAGAAGCGGGGGCCGGCACCGGATGAACAGGATACCCCCCTCTAGCCGGCAACCTGGCCCACGCCGGAGCCTCAGTAGACCTAGCAATCTTCTCCCTGCACTTAGCAGGGATTTCTTCAATCTTAGGGGCAATCAACTTTATTACAACAGGAATCAACATAAAACCACCTGCCCTAT | *Passer domesticus* | KM078784.1 |
|  |  |  |  |  |
| 2015-237T | MERL | TCTACACCTAGTCTTACTTATCTGATCAATACTATGCAACCTGCGCTCTCTTGGGAGACGACCTATACCTACTACTTTCGTAATAATCTTTTTTATAGTTATGCCAATTATGATTGGAGGATTTGGAAACTGACTAGTTCCTCTAATAATTGGAGCACCTGACATAGCATTCCCACGAATGAATAATATAAGCTTCTGACTTCTACCCCCATCCTTTCTTCTTCTACTAGCCTCCTCTACAGTAGAGGCCGGAGTGGGAACAGGATGAACTGTGTATCCCCCCCTGGCTGGTAATTTAGCCCACGCCGGGGCGTCAGTTGATTTAGCAATTTTCTCCCTACATCTAGCAGGAATTTCATCTATCCTAGGAGCAATCAATTTCATTACCACTGCAATTAACATAAAACCACCTGCCCTATCA | *Sitta canadensis* | HM033802.1 |
|  |  |  |  |  |
| 2015-034B | MERL | ACGGCCCATGCCTTCGTAATAATCTTCTTTATAGTTATGCCCATTATGATCGGAGGGTTCGGAAACTGACTAGTCCCACTGATAATCGGAGCCCCAGACATAGCATTCCCACGAATAAACAACATAAGCTTCTGACTACTTCCCCCATCCTTCCTTCTACTCCTAGCATCCTCTACCGTAGAAGCAGGGGTTGGACAGGATGAACAGTATACCCCCCACTAGCTGGTAACTTAACCCATGCCGGAGCCTCAGTTGACTTAACAATCTTCTCCCTACACCTAGCTGGTATCTCTTCAATCCTAGGAGCAATTAACTTTATTACCACAGGAATCAATATAAAACCACCTG | *Haemorhous mexicanus* | JN850723.1 |
|  |  |  |  |  |
| 2015-034T | MERL | ACGACCAAGTATATAACGTAATCGTCACGGCCCATGCTTTCGTAATAATCTTTTTTATAGTTATGCCCATTATGATTGGAGGATTTGGAAACTGACTAGTTCCTCTAATAATTGGAGCACCTGACATAGCATTCCCACGAATGAATAACATAAGCTTCTGACTTCTACCCCCATCCTTCCTTCTTCTACTAGCCTCCTCTACAGTAGAGGCCGGAGTAGGAACAGGATGAACTGTGTATCCTCCCCTGGCTGGTAATTTAGCTCACGCCGGGGCGTCAGTTGATTTAGCAATTTTCTCCCTACATCTAGCAGGAATTTCATCTATCCTAGGAGCAATCAATTTCATTACCACTGCAATTAACATAAAA | *Sitta canadensis* | KJ467141.1 |
|  |  |  |  |  |
| 2015-055B | MERL | AACGTAGTCGTCACGGCCCATGCTTTCGTAATAATTTTCTTTATAGTTATGCCCATCATAATTGGAGGATTCGGAAACTGACTAGTCCCTCTGATAATCGGAGCCCCAGACATAGCATTCCCACGAATAAACAACATAAGCTTCTGACTACTCCCACCATCGTTCCTTCTCCTTCTAGCGCCCTCCACGGTTGAAGCAGGAGTAGGTACAGGCTGAACAGTGTACCCCCCACTAGCCGGTAACCTGGCCCACGCCGGAGCCTCAGTCGACCTCGCAATCTTCTCTCTACACCTAGCCGGTATTTCCTCAATCCTAGCGCAATCAACTTCATTACAACAGCAATTAACATGAAACCACCTGCCCTATCA | *Setophaga petechia* | JN850722.1 |
|  |  |  |  |  |
| 2015-055T | MERL | CGTCACGGCCCATGCTTTCGTAATAATTTTCTTTATAGTTATACCCATCATAATTGGAGGATTCGGAAACTGACTAGTTCCCCTAATAATCGGAGCCCCAGACATAGCATTCCACGATAAACAACATAAGCTTCTGACTACTCCCACCATCGTCCTTTCCTTTAGCGCCTCCACGGTTGAAGCGGAGTAGGTACAGGCTGAACAGTGACCCCCCACTAGCCGGAACCTGGCCCACGCCGGAGCCTCAGTCGACCTGGCAATCTTCTCTCTACACCTAGCCGGTATTTCCTCAATCCTAGGAGCAATCAACTTCATTACAACAGCAATTAACATGAAACCACCTGCCCTATC | *Setophaga petechia* | JN850722.1 |
|  |  |  |  |  |
| 2015-334B | MERL | GCTCTCCTAGGTGACGACCAAATCTACAACGTGGTTGTCACCGCCCATGCTTTCGTAATAATCTTCTTCATAGTTATACCAATTATGATCGGAGGGTTCGGAAACTGACTAGTCCCCCTAATAATCGGAGCCCCAGACATAGCATTCCCCCGAATAAACAACATAAGCTTTTGACTCCTTCCCCCATCCTTCCTTCTCCTCCTAGCCTCCTCCACAGTAGAAGCTGGGGCAGGGACAGGTTGAACCGTCTACCCACCCCTCGCCGGCAACCTAGCACACGCAGGGGCTTCAGTAGACTTGGCCATTTTCTCCCTACACTTAGCAGGGATCTCCTCAATCCTAGGGGCCATCAACTTCATCACAACAGCAATCAACATAAAACCACCTGCCCTAT | *Turdus migratorius* | KJ909198.1 |
|  |  |  |  |  |
| 2015-334T | MERL | CTCTACTAGGAGACGACCAAATCTACAACGTAGTAGTTACCGCTCACGCCTTCGTAATAATCTTCTTTATGGTTATGCCTATCATAATCGGAGGGTTCGGAAACTGACTAGTGCCCCTAATAATCGGAGCCCCAGACATAGCATTCCCTCGAATAAACAACATAAGCTTCTGACTTCTCCCCCCATCCTTCCTACTCCTCCTAGCCTCCTCCACAGTCGAAGCAGGGGTTGGAACAGGCTGAACCGTCTACCCCCCTCTGGCTGGCAACCTCGCCCACGCTGGGGCCTCAGTAGACCTCGCTATCTTCTCCCTCACCCTGGCAGGGATCTCCTCAATCCTAGGGGCTATTAACTTCATCACACCCGAAATAAACAAAAAACCACCTGCCCTA | *Sturnus vulgaris* | GU571639.1 |
| **Sample ID** | **Control** | **Recovered Sequence** | **Prey detected on swab** | **BLAST Accession #** |
| Control 1 | YEWA | CCATCTCCTAGCCTCCTCTTCGGCAGACTAGGCCACCCGGAGCCCTTCTGGGAGACGACCAAGTCTATAATGTAGTCGTCACGGCCCATGCCTTCGTAATAATTTTCTTTATAGTTATGCCAATTATAATTGGAGGATTCGGAAACTGACTAGTCCCTCTAATAATCGGAGCCCCAGACATAGCATTCCCACGAATAAACAACATAAGCTTCTGACTACTCCCACCATCGTTCCTTCTCCTTCTAGCGTCCTCCACGGTTGAAGCAGGAGTAGGTACAGGCTGAACAGTGTACCCCCCACTAGCCGGTAACCTGGCCCACGCCGGAGCCTCAGTCGACCTCGCAATCTTCTCTCTACACCTAGCCGGTATTTCCTCAATCCTCGGAGCAATCAACTTCATTACAACAGCAATTAACATGAAACCACCTGCCCTA | *Setophaga petechia* | HM033412.1 |
|  |  |  |  |  |
| Control 2 | OCWA | CACTCCCCTAGCCTCTTTATCCGGCAGACTAGGCAACCCGGAGCCCTTCTGGGAGACGACCAAGTCTACAATGTAGTTGTCACGGCCCATGCTTTCGTAATAATTTTCTTTATAGTCATACCGATTATAATCGGAGGATTCGGAAACTGACTAGTTCCCCTAATAATCGGAGCCCCAGACATAGCATTCCCACGAATAAACAACATAAGCTTCTGACTACTCCCACCATCATTCCTTCTCCTACTAGCATCCTCCACAGTTGAAGCAGGTGTCGGCACAGGTTGAACAGTGTACCCTCCACTAGCTGGCAACCTAGCCCACGCCGGAGCCTCCGTCGACCTTGCAATTTTCTCTCTACACCTGGCTGGTATTTCCTCAATCCTCGGGGCGATCAACTTCATTACAACAGCAATCAACATGAAACCACCTGCCCTATC | *Vermivora celata* | FJ236284.1 |
|  |  |  |  |  |
| Control 3 | SWTH | CCTTGCCCTAGCCTTCTATCCGGCAGACTAGGCCACCAGGCGCACTACTAGGTGACGACCAAATCTACAATGTAGTTGTCACCGCCCACGCCTTCGTAATGATTTTCTTTATAGTTATGCCAATCATGATTGGGGGGTTCGGAAACTGGCTAGTCCCATTAATAATCGGAGCCCCAGACATAGCATTCCCCCGAATAAACAACATAAGCTTCTGACTTCTCCCACCATCATTCCTCCTTCTCCTAGCCTCCTCCACAGTAGAAGCAGGAGCAGGAACAGGATGGACCGTCTATCCACCCCTCGCTGGCAACCTAGCACACGCAGGAGCCTCAGTCGACCTAGCTATTTTCTCCCTCCACTTAGCAGGAATCTCCTCAATCCTAGGGGCCATCAATTTCATTACTACAGCAATCAACATAAAACCACCTGCCCT | *Catharus ustulatus* | HM033284.1 |

**Supplementary Table 1:** Listed are recovered COI sequences from swabs that collected prey DNA from migrating raptor beaks and talons. Also included are the COI sequences of control songbird DNA. Raptor and control species names are displayed in 4-letter banding codes. Accession # from BLAST searches are also included for the top result with the closest match. Refer to Table 1 for details on % match and likelihoods that match is not random chance.
